# Clinical, Molecular, and Epidemiological Analysis of Dengue Cases during a Major Outbreak in the Midwest Region of Minas Gerais, Brazil

**DOI:** 10.1155/2014/276912

**Published:** 2014-07-10

**Authors:** Juliana Cristina Duarte Braga, Leandro César da Silva, Jacqueline Domingues Tibúrcio, Mirna de Abreu e Silva, Lailah Horácio Sales Pereira, Karina Rocha Dutra, Jaqueline Maria Siqueira Ferreira, Débora de Oliveira Lopes, Luciana Lara dos Santos

**Affiliations:** ^1^Universidade Federal de São João del Rei (UFSJ), Avenida Sebastião Gonçalves Coelho, 400 Chanadour, 35501-296 Divinópolis, MG, Brazil; ^2^Secretaria de Vigilância em Saúde de Divinópolis, Minas Gerais Street, 900 Centro, 35500-007 Divinópolis, MG, Brazil

## Abstract

This study aims to perform the first molecular and clinical-epidemiological analysis of dengue cases in Divinopolis, MG, Brazil. Data from 4,110 cases of dengue were accessed and 190 clinical samples were collected for molecular analyses. In this study, 2.7% of the men and 3.0% of the women were admitted to hospital. There was no association between gender and hospital admission. The symptoms observed in this study are according to the Health Ministry, but fever was present in 82.2% and not in 100% of cases. The chance of hospital admission was 1.55 higher in patients with any kind of bleeding (334) and 2.4% of individuals without bleeding were also hospitalized due to other warning signs. In the molecular analyses, 23% of the samples were positive for DENV. DENV-2 and DENV-3 were identified in 2010, DENV-3 in 2011, DENV-1 in 2012, and DENV-1 and DENV-4 in 2013. DENV detection was possible in samples with only one day of symptoms. This first report of dengue data in Divinópolis provided more insight into the viral types and effects of disease in the city, confirming the need for caution in assessing cases of suspected dengue and for revision of the criteria proposed by the Health Ministry to classify cases of the disease.

## 1. Introduction

Dengue is a disease caused by four antigenically distinct but genetically related virus serotypes that cause dengue:* Dengue virus* (DENV 1–4) [1]. It is transmitted by the* Aedes aegypti* mosquito with clinical presentation of the disease ranging from mild forms, such as dengue fever (DF), to serious and even fatal forms. In the mild form of the disease, clinical manifestations include fever, headache, prostration, arthralgia, retroorbital pain, nausea, rash, itchy skin, and others. The main severe form of dengue is dengue hemorrhagic fever (DHF), characterized by bleeding tendency, thrombocytopenia, and plasmatic effusion, which can progress to circulatory failure, characterizing dengue shock syndrome (DSS), and death [2–6].

The incidence of dengue has grown worldwide in recent decades. The number of people at risk is about 2.5 billion. A recent study estimates there to be 390 million dengue infections every year, of which 96 million manifest any level of clinical or subclinical severity [7]. In 2007, there were over 890,000 dengue cases in America, of which 26,000 were DHF. The disease is endemic in over 100 countries in Africa, Americas, Eastern Mediterranean, Southeast Asia, and Eastern Pacific. Southeast Asia and Eastern Pacific are the most seriously affected. Before 1970, only nine countries had experienced DHF epidemics, a number that has increased more than four times in 1995 [4]. Brazil has experienced several epidemics of dengue [8].

From 2001 to October 2013, there were 13,955 confirmed cases of dengue in Divinópolis, midwest of Minas Gerais (MG), Brazil, of which 4,350 were in 2010 [9]. Since the first case reported of dengue, eight deaths were registered. During this period, viral isolation detected the circulation of DENV-1 and DENV-2 serotypes in 2001; DENV-3's emergence in 2002; and DENV-1 and DENV-3 in 2003 [10]. The viral typing for the years 2010, 2011, 2012, and 2013 was performed in this work.

The increase of dengue cases accompanied by severe forms of disease and the detection of three dengue serotypes in the city show that it is necessary to study the clinical, molecular, and epidemiological characteristics of dengue cases during a major dengue outbreak in Divinopolis, MG.

## 2. Methods

### 2.1. Clinical-Epidemiological Analysis

The information system for notifiable diseases and the local epidemiological surveillance database were used to access the data of patients with DF. Dengue fever cases were confirmed by epidemiological criteria or serological tests by different laboratories for IgM/IgG detection using an IgM antibody-capture enzyme-linked immunosorbent assay (MAC-ELISA) (PanBio, Brisbane, Australia) or rapid chromatographic immunoassay kit for detection of Dengue IgG/IgM antivirus (WAMA Diagnóstica, SP, Brasil).

Forms from the information system for notifiable diseases consist of general data (city, date of notification, and health care institution), individual data (identification, gender, race, and education), residence (address, urban, or rural), clinical manifestations (fever, headache, prostration, arthralgia, myalgia, exanthema, vomiting, diarrhea, bleeding, retroocular, and abdominal pain), and laboratory to qualify the case. The data analyzed in this study include gender, age, clinical manifestations, days of the symptoms, results of serological tests, and data about hospital admission. Cases without fever but with many other symptoms described above were considered suspicious once atypical dengue cases were previously described in the country.

The research data were entered in duplicate, validated, and processed in Microsoft Office Excel 2007. Statistical Package for the Social Sciences (SPSS) 14.0 was used for statistical analysis. Univariate analyses between categorical variables, sex and hospital admission and bleeding and hospital admission, were performed.

Univariate analyses between days with symptoms and hospitalization and days with symptoms and bleeding were performed as well. The chi-square, odds rates, and Mann Whitney test were used to estimate the odds ratio and median with a significance level of 5%.

There were 5,032 reported cases of dengue in 2010 in the municipality, but only cases confirmed by laboratory test or epidemiological criteria were included. Of the total, 699 forms were discarded without confirmation of the disease and some others discarded by incomplete filling or by typographical errors. Therefore, this study was performed with 4,110 cases.

### 2.2. Molecular Analysis

For molecular analyses, 190 samples from patients with suspected dengue were collected from 2010 to 2013. The collection was performed from January to March of each year at the Hospital São Judas Tadeu or at the Centro Municipal de Apoio à Saúde (CEMAS), both in Divinopolis, MG. The samples were collected from patients with less than 7 days of symptoms after the consent on the research project. The participants answered a questionnaire about symptoms present. This study was approved by the Research's Ethics Committee of the Universidade Federal de São João del Rei, under the identification 012/2010.

Plasma for molecular analyses was obtained after centrifugation of 3 to 5 mL of whole blood and stored at −80°C until RNA extraction. The RNA extraction was performed with the QIAamp viral RNA minikit (QIAGEN, USA) and the RNA fractionated and stored at −80°C. For the detection and typing of the DENV serotypes, primers described by Lanciotti et al. [11] were used. After linearization with reverse primer, the RNA was subjected to RT-PCR using the following conditions: 1 h at 42°C and 15 min at 70°C using buffer RT enzyme (5x), dNTP (1 mM), and RT enzyme (100 U).

DENV were detected using 5 uL of cDNA, 1.25 uL of Taq DNA polymerase, Taq Buffer (5x), 2 mM MgCl_2_, 10 pmoles of each primer, and 100 uM of dNTP. Nested PCR was performed for viral typing using 5 *μ*L from the dilution of the amplified product (1 : 10 in sterile water). The PCRs for detection and DENV typing were performed under the same conditions: 5 min at 94°C, 30 cycles of 94°C for 30 s, 55°C for 30 s, and 72°C for 30 s. Fragments were visualized on 8% polyacrylamide gels stained with silver nitrate.

## 3. Results

### 3.1. Most Frequent Symptoms

**Figure 1:**
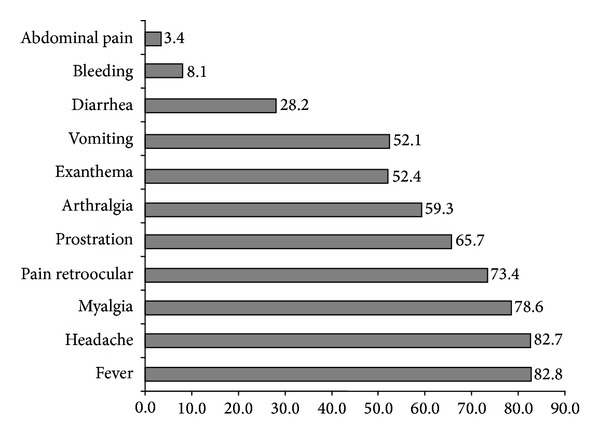
Most frequent symptoms observed in patients with dengue in the major outbreak of the Divinopolis, MG.

The most frequent symptoms present in the notification form from the information system for notifiable diseases are described in Figure 1.

Prostration, arthralgia, exanthema, vomiting, diarrhea, bleeding, retroocular and abdominal pain, headache, and fever may be observed. In this analysis, the most common symptoms were fever (82.8%), followed by headache (82.7%), myalgia (78.6%), and pain retroocular (73.4%).

Of the total subjects studied, 206 (5.0%) reported the presence of some other symptoms besides those described in the information system for notifiable diseases form. The most frequent were petechiae at 44.7% (92/206) and itching at 22.8% (47/206).

In this study, 334 (8.1%) individuals had bleeding. Information about type of bleeding was present only for 67 (20.0%) patients. Epistaxis (50.7%), gingival bleeding (25.3%), and metrorrhagia (19.4%) were the most frequent types. Even among individuals who reported bleeding, six of them (8.9%) had more than one type.

### 3.2. Association between Bleeding and Hospital Admission

Of the 4,110 individuals analyzed, 40.2% (1,651) were male and among them 2.7% (44/1,651) were hospitalized. Among the women (59.8% of the sample), 3.0% were admitted to hospital. However, chi-squared testing was used to verify that there was no association between gender of the patient and hospital admission (*P* = 0.517).

Individuals who had been admitted to hospital showed on average five days of symptoms compared with three days of symptoms of those not admitted. In the statistical analyses, the difference between the median number of the days of symptoms in patients who were or were not admitted to hospital was significant (*P* < 0.01).

In the association analysis of the hospital admission and bleeding, data were available for 110 individuals that were admitted to hospital and for 3,222 individuals that were not admitted. So, the correlation analysis between hospital admission and bleeding was restricted to 3,432 patients. The results can be visualized in Table 1.

Among 3,432 patients, 9.7% had any kind of bleeding (334/3,432), and, among these, 10.5% (35/334) were hospitalized. On the other hand, from 90.3% with no bleeding (3,098/3,432), 2.4% were admitted to hospital (75/3,098) but other warning signs as vomiting and abdominal pain were present in 63% of these individuals.

The median number of days with symptoms was four in people who had bleeding and three in people without bleeding. This difference was statistically significant (*P* < 0.05).

### 3.3. Serological and Molecular Diagnosis

Only 23% (946) of cases were confirmed by serological tests and the other 70,1% (2,880) were confirmed by epidemiological criteria. This information was not available for 6,9% (284) individuals.

Of the 190 blood samples collected from patients with suspected dengue from 2010 to 2013, 23% were positive for DENV by molecular tests.

**Figure 2:**
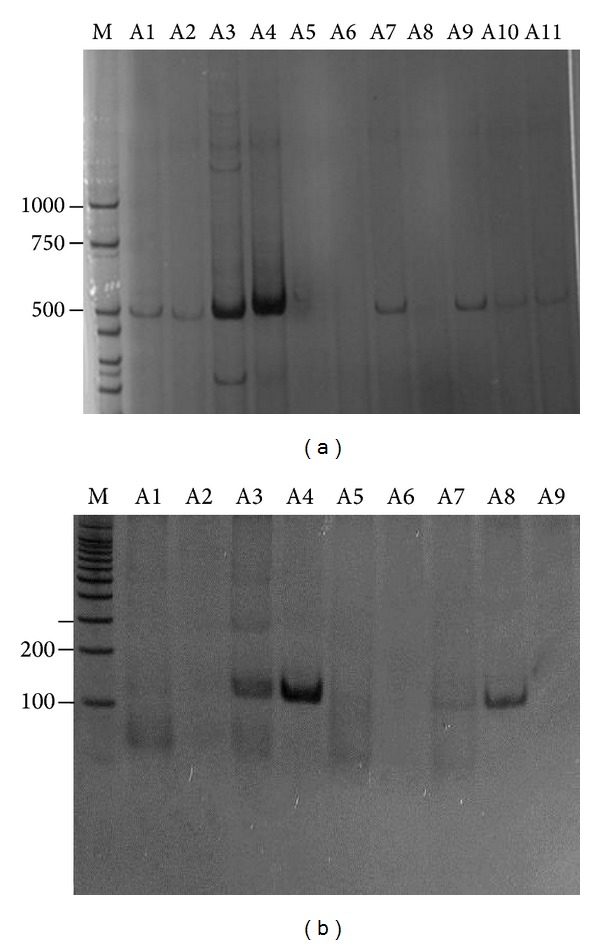
*Dengue virus* amplification by RT-PCR from blood samples patients. (a) Electrophoretic profile of DNA fragments (511 pb) corresponding to positive samples. A5, A6, A8: negative samples for* Dengue virus*. (b) Electrophoretic profile from the nested PCR for viral typing. A3, A4, A7, A8: DENV-2 fragment of 119 pb. PCR products were fractioned by 8% PAGE and visualised by silver staining. M: molecular size markers (bp).

In 2010, of the 82 blood samples collected, 22 were positive in the molecular test. The viral typing performed by nested PCR corresponded to DENV-2. This profile was found in 21 positive samples (Figure 2). Just one sample was positive for DENV-3. Among 22 positive cases detected by molecular diagnosis, fever was not reported by nine of these patients.

In subsequent years, the typing of DENV has continued with DENV-3 detected in the single positive sample in 2011 from 32 samples collected and DENV-1 in one positive sample from 6 suspected cases collected in 2012. The years 2011 and 2012 had just 35 and 27 confirmed dengue cases by serological test. In 2013, 5,998 cases were confirmed until October of this year with DENV-1 detected in 20 positive samples from 70 suspected cases collected.

Molecular diagnosis allowed the detection of DENV in samples with only one day of symptoms. Positive cases in the years analyzed were distributed as follows: five cases with one day of symptoms, 13 cases with two days of symptoms, 16 cases with three days of symptoms, five cases with four days of symptoms, one case with five days of symptoms, and one case with 6 days of symptoms. This information was missing for three patients. One patient had a negative serology but a positive molecular diagnosis and three other patients had positive serology but negative molecular diagnoses.

## 4. Discussion

Dengue fever, which is a public health problem, is part of a scenario that consists of two factors of concern, since the presentation clinic is nonspecific: (i) most of the dengue cases go unreported; (ii) during epidemic periods, erroneous notifications may occur in some individuals with similar clinical manifestations caused by other viral agents. The scarcity of financial resources and specific laboratory for dengue diagnosis contribute to these problems [2, 12, 13]. Thus, clinical, molecular, and epidemiological studies of dengue cases during epidemic periods are important to review the proposed criteria for classification of dengue frames of the Health Ministry, to emphasize the need for surveillance and the need for caution of health professionals in evaluating suspected cases [6].

The symptoms observed in this study are consistent with the protocol of the Department of Health Surveillance, but not all patients had fever and this data was confirmed by epidemiological and molecular evaluation. According to the Health Ministry, dengue infection ranges from asymptomatic forms to severe cases, but fever has been used together with other symptoms to classify the disease. Furthermore, in this present study, RT-PCR identified nine positive cases without fever among the samples collected in 2010. Our results corroborate other published article, which reported in their study about higher accuracy of a predictive model for early dengue diagnosis, including clinical and laboratory features better than the current WHO guidelines for suspected dengue [14].

In the association analysis of hospital admission and bleeding (Table 1), the chance of hospital admission was 1.55 (1.13 to 1.97) higher in patients with any kind of bleeding than those without bleeding. Regarding admissions for dengue infection, it is important to note that there are criteria recommended by the Brazilian Ministry of Health, as the presence of warning signs, refusal in food intake and liquids, respiratory compromise, chest pain, difficulty breathing, decreased breath sounds or other signs of severity, and platelets <20.000/uL regardless of hemorrhagic manifestations [6]. Of the 334 patients with bleeding, 90% were not hospitalized. In 2010, according to the recommendations of the Ministry Health, inexpressive bleeding did not require hospitalization which may explain the small number of hospitalized individuals even with bleeding. Any bleeding in mucosa was included as a warning sign only after that year. Among the 75 (2.4%) individuals who were admitted in the hospital without bleeding, at least 63% presented other warning signs as vomiting, but records about other criteria described above were not assessed in this study and probably justify this clinical practice.

In spite of the large number of individuals hospitalized in 2010, 37 cases were classified by the Department of Health Surveillance of Divinopolis as dengue with complications, with two deaths and four cases of dengue hemorrhagic fever [9].

The results found in this study are consistent with the literature showing patients with an average of 5 days of symptoms being admitted more to hospital compared with 3 days of symptoms of those not admitted. Moreover, the number of days of symptoms on average observed for those individuals with bleeding was also higher. Usually the critical phase can be observed on days 3–7 of the illness after the febrile phase that is marked mainly by dehydration and high fever. The critical phase can include shock from plasma leakage, severe hemorrhage, and organ impairment [2, 15]. Therefore, monitoring for warning signs and other clinical parameters is crucial to recognizing progression to the critical phase.

According to data released by the Department of Health Surveillance, reported cases of dengue in Brazil in 2008 by sex are higher in women than in men. This fact is also confirmed in this study and is easily understood since the vector is commonly residential. However, when evaluating statistically the number of the hospital admission in relation to sex, there was no significant association (*P* = 0.517). Contrastingly, a study reported that male sex was predominant (65.2%) among dengue cases but was not among nondengue patients (47.9%) [16].

Dengue fever can easily be confused with nondengue infections, particularly in nonepidemic situations. Identification of virus, viral RNA, and viral antigen and the detection of an antibody response are preferable for the diagnosis of dengue [12]. If samples are collected after day 5 of illness, reliable serological tests can be performed in a reference laboratory. Serological assays may be used to determine the extent of outbreaks. However, during major epidemics most cases are epidemiologically confirmed, since it would be impossible to test all of them in a reference laboratory. In this study, this fact accounts for the low percentage at 23% of serological tests performed in 2010.

In 2010, to identify the serotype of virus circulating in the municipality, molecular diagnosis was performed for 82 patients with suspected dengue. DENV-2 and DENV-3 were found in 22 positive samples. The epidemic of 2010 can be due to the introduction of DENV-2, which had not circulated in the municipality for many years. DENV-1 and DENV-2 were isolated in samples collected in 2001 in Divinopolis, and DENV-3 was the major serotype found in the 2002 epidemic in Brazil, which resulted in a higher incidence of hemorrhagic cases leading to death [14]. According to the Epidemiological Surveillance of Divinopolis, serological identifications during 2002 confirmed the cocirculation of DENV-3 and DENV-1. The prevalence rates decreased from 2004 onwards, possibly due to the induction of partial group immunity. The number of reported cases increased gradually from 2004 to 2009 years in which studies identifying viruses were not performed. In 2010, probably with the reintroduction of DENV-2, a new epidemic has occurred. In 2011 and 2012, the number of cases has decreased sharply with 35 and 27 confirmed cases, respectively. DENV-3 was identified in 2011 and DENV-1 in 2012 in only positive samples by RT-PCR. In 2013, 1,476,917 suspected dengue cases were reported in Brazil. This number is almost three times higher than the same period in the last year. The increase in the number of cases is primarily due to proliferation of DENV-4; a new strain of the virus reached a population still immunologically unprotected which led to an epidemic. In this same year, the largest epidemic in the history of the Divinopolis took place with 6,015 cases notified and DENV-1 and DENV-4 detected in samples analyzed by molecular test. DENV-1 was isolated in Divinopolis 12 years ago and in only a positive sample in 2012, and DENV-4 was isolated for the first time in 2013, which could explain the epidemic in the municipality.

Molecular diagnosis allowed the detection of DENV in samples with only one and two days of symptoms. Molecular diagnosis has an inverse relationship compared to diagnosis made by serology [4]. The serological test requires the production of antibodies, which takes about seven days of symptoms. The test sensitivity falls after the first day of symptoms. The sensitivity of molecular diagnosis falls as the days of symptoms increase because the antibodies begin to act against the virus and there is a decrease in viral load. This relationship was also observed in this present study where one patient had a negative serology but a positive molecular diagnosis. This patient had two days of symptoms and most likely serological testing was not done at the right time accounting for the discordant results. Nevertheless, three patients were found with positive serology but negative molecular diagnosis. These three patients reported six days of symptoms. The discordant results may be due to the viral load reduction as the days of symptoms increase and the bias that may be incorporated by reports that do not correspond to the days of exact symptoms of the patient.

## 5. Conclusion

The results of this study confirm the need to review the criteria proposed by the Health Ministry to classify cases of dengue since fever is not present in all of cases and is classified as the first manifestation of the infection. The health professionals should be cautious in evaluating suspected cases since the presentation clinic of the disease is nonspecific. The typing viral in these last years in Divinopolis reflects what takes place in the rest of the country being the DENV-4, for the first time, confirmed in clinical samples from 2013 in the municipality. Future sequencing and phylogenetic analysis of the RT-PCR amplified virus will be performed in positive samples that were found in Divinopolis, mainly to increase the knowledge about the DENV-4 once a distinct genotype was described recently in Brazil, increasing the potential of outbreaks in the country [17].

## Figures and Tables

**Table 1:** Correlation analysis between bleeding and hospital admission.

	Admitted	Not admitted	
Bleeding			
yes			
*n*	35	299	334
%	10.5%	89.5%	100.0%
no			
*n*	75	3,023	3,098
%	2.4%	97.6%	100.0%

Total			
*n*	110	3,322	3,432
%	3.2%	96.8%	100.0%
